# Relations of microbiome characteristics to edaphic properties of tropical soils from Trinidad

**DOI:** 10.3389/fmicb.2015.01045

**Published:** 2015-09-30

**Authors:** Vidya de Gannes, Gaius Eudoxie, Isaac Bekele, William J. Hickey

**Affiliations:** ^1^Department Food Production, University of the West IndiesSt. Augustine, Trinidad and Tobago; ^2^O.N. Allen Laboratory for Soil Microbiology, Department Soil Science, University of Wisconsin-MadisonMadison, WI, USA

**Keywords:** soil, bacteria, fungi, archaea, Illumina

## Abstract

Understanding how community structure of *Bacteria, Archaea*, and *Fungi* varies as a function of edaphic characteristics is key to elucidating associations between soil ecosystem function and the microbiome that sustains it. In this study, non-managed tropical soils were examined that represented a range of edaphic characteristics, and a comprehensive soil microbiome analysis was done by Illumina sequencing of amplicon libraries that targeted *Bacteria* (universal prokaryotic 16S rRNA gene primers), *Archaea* (primers selective for archaeal 16S rRNA genes), or *Fungi* (internal transcribed spacer region). Microbiome diversity decreased in the order: *Bacteria* > *Archaea* > *Fungi*. Bacterial community composition had a strong relationship to edaphic factors while that of *Archaea* and *Fungi* was comparatively weak. Bacterial communities were 70–80% alike, while communities of *Fungi* and *Archaea* had 40–50% similarity. While each of the three component communities differed in species turnover patterns, soils having relatively similar bacterial communities also housed similar archaeal communities. In contrast, the composition of fungal communities had no correlation to bacterial or archaeal communities. Bacterial and archaeal diversity had significant (negative) correlations to pH, whereas fungal diversity was not correlated to pH. Edaphic characteristics that best explained variation between soils in bacterial community structure were: total carbon, sodium, magnesium, and zinc. For fungi, the best variables were: sodium, magnesium, phosphorus, boron, and C/N. Archaeal communities had two sets of edaphic factors of equal strength, one contained sulfur, sodium, and ammonium-N and the other was composed of clay, potassium, ammonium-N, and nitrate-N. Collectively, the data indicate that *Bacteria, Archaea*, and *Fungi* did not closely parallel one another in community structure development, and thus microbiomes in each soil acquired unique identities. This divergence could in part reflect the finding that unknown factor(s) were stronger than edaphic characteristics in shaping fungal and archaeal communities.

## Introduction

Soils are habitats for a diversity of microbes with *Bacteria, Archaea*, and *Fungi* all important in contributing functions that sustain these ecosystems. Application of next generation sequencing (NGS) technologies (e.g., 454 Pyrosequencing or Illumina) has greatly advanced our understanding of soil microbial community composition, but the majority of those studies have focused on communities of only a single microbial component, i.e., *Bacteria* (Acosta-Martinez et al., 2008; Lauber et al., 2009; Will et al., 2010; Nacke et al., 2011; Fierer et al., 2012; Uroz et al., 2013), *Fungi* (Buée et al., 2009; Lentendu et al., 2011; Orgiazzi et al., 2013; Schmidt et al., 2013), or *Archaea* (Bates et al., 2011; Singh et al., 2012; Tripathi et al., 2013; Richter et al., 2014). A limited number of NGS-based investigations have concurrently examined communities of bacteria and fungi (Rousk et al., 2010; Zinger et al., 2011; Lienhard et al., 2014), while archaea have been examined to the extent that archaeal 16S RNA genes amplify along with those of bacteria with universal prokaryotic PCR primers (Roesch et al., 2007; Eilers et al., 2012). Examination of all three microbial groups concurrently is needed for a comprehensive assessment of the soil microbiome.

The emergence of NGS approaches has been particularly important for soil microbiome studies, which aim to elucidate relationships between the phylogenetic structure of microbial communities and soil characteristics. To date, NGS-based soil microbiome investigations have mostly focused on relating edaphic properties to single components of the soil microbiome (i.e., bacteria, fungi or archaea), and pH is often identified as a predominant factor affecting bacterial and archaeal communities, but not fungi (Acosta-Martinez et al., 2008; Lauber et al., 2009; Nacke et al., 2011; Fierer et al., 2012). Impacts of pH on soil microbial communities were well-established long before the advent of NGS (Bååtha and Anderson, 2003; Högberg et al., 2006; Rousk et al., 2010), but are difficult to interpret because of the many direct and indirect effects pH may exert. Moreover, although thorough physicochemical characterization of soils is an essential element in soil microbiome investigations, the type and range of these properties examined in such studies varies widely, and complicates identification of unifying themes. For fungi, impacts of edaphic properties on community structure may be subordinate to plant type and diversity (Opik et al., 2009; Lin et al., 2012; Mouhamadou et al., 2013) perhaps reflecting important fungal life styles as plant symbionts, plant pathogens and decomposers of plant polymers. However, most NGS studies of soil fungi are slanted toward woodlands and analysis of ectomycorrhizal fungi (Jumpponen et al., 2010; Opik et al., 2010; Tedersoo et al., 2010; Lin et al., 2012), so the relative importance of vegetation vs. edaphic properties on these organisms is still uncertain. Archaeal communities in soil have been under-investigated, as PCR primers with archaeal selectivity greater than that of universal prokaryotic primers have typically not been applied in soil microbiome analyses.

While edaphic properties are a primary environmental filter affecting soil microbiome structure, comprehensive analyses of all microbiome components integrated with thorough soil characterization is lacking. This information is needed to gain insight into a variety of fundamental processes, such as those controlling soil microbiome turnover. Understanding edaphic controls on turnover, the change in microbiome composition as a function of soil properties, can help answer important questions about the interactions between the soil microbes and the environment that they inhabit. For example, is there a hierarchy of soil-microbiome responses, with some components more strongly controlled by soil properties than others (e.g., bacteria vs. fungi) and, if so, what are the edaphic factors that affect microbial communities differentially? Elucidating edaphic controls on soil microbiome turnover could also help in understanding the extent to which microbiome components covary as a function of soils, and in unraveling connections between microbiome composition and key ecosystem services.

The present study focused on non-managed tropical soils representing a range of edaphic characteristics. There is comparatively little information about soil microbiomes in tropical and subtropical regions, especially the fungal and archaeal components. The soils examined in this study, represented a range of pH, a range of edaphic characteristics, and a comprehensive assessment of the microbial communities in those soils was obtained by Illumina sequencing of amplicon libraries of either 16S ribosomal RNA genes for bacteria and archaea, or internal transcribed spacer (ITS) regions for fungi. Two hypotheses were tested. First, pH was a master variable affecting diversity and that composition and diversity of all three groups would be correlated with that characteristic. Second, each group would have a similar set of edaphic factors that correlated with differences in composition and diversity. Third, all three components of the soil microbiomes would covary in alpha- and beta-diversity. The objectives were: (1) To determine how the community structure of bacteria, archaea and fungi varied, and (2) To identify the edaphic factors that correlated with variation in community structure for each microbial group.

## Materials and methods

### Soil sampling, DNA extraction, and sequencing

Soils used in this study was previously described (de Gannes et al., 2014). All nine soils were sampled from non-managed locations vegetated primarily by grasses *viz.* Elephant grass (*Pennisetum purpureum*) and Fowl Foot grass (*Eleusine indica*) as the predominant species. The exception was the Arena sandy loam which was sampled from a preserve of seasonal evergreen forest *viz*. Crappo (*Carapa guianensis*), Balata (*Manilkara balata*), and Manhoe (*Sterculia caribaea*). The sampling strategy adopted was similar to other studies examining biogeography of soil microbial communities (Fierer and Jackson, 2006; Roesch et al., 2007; Bates et al., 2011, 2013) and employed compositing of soil samples at sites (soil types) to focus on comparisons between sites rather than within sites. In an effort to minimize variability from the DNA extraction process, 12 extractions were prepared from each soil in batches of 0.25 g, which were pooled to generate three 1 g-equivalent samples. A 1 uL aliquot from each of the 1 g-equivalent extracts was used for generation of bacterial, archaeal and fungal amplicon libraries.

Bacterial libraries were created with universal prokaryote primers 515F and 806R that targeted the V4–V5 region of the 16S rRNA gene (Caporaso et al., 2010) while archaeal libraries were developed by using primers archaea349F and archaea806R, which have selectivity for the V3–V4 region of archaeal 16S rRNA genes (Takai and Horikoshi, 2000). For both of these groups, amplicons were generated following PCR protocols described in the work referenced as the source of primers. Fungal libraries were created with primers ITS1F (Gardes and Bruns, 1993) and ITS4 (White et al., 1990) following the PCR protocol described by Manter and Vivanco (2007). Region specific primers were modified to add Illumina adapter overhang nucleotide sequences to the amplicons. Following initial amplification, library size was verified on an Agilent DNA1000 chip, and cleaned using a 1X volume of Mag PCR clean-up beads (Axygen Biosciences, Union City, CA). Illumina dual indexes and sequencing adapters were added using the forward primer 5′AA TGATACGGCGACCACCGAGATCTACAC[55555555]ACACTCTTTCCCTACACGACGCTCT TCCGATCT-3′, and reverse primer: 5′-CAAGCA GAAGACGGCATACGAGAT[77777777]GTGACTGGAGTTCAGACGTGTGCTCTTCCGAT CT-3′. In the forgoing primer sequences the bracketed regions were equivalent to the Illumina Dual Index adapters D501–D508 and D701–D712, respectively. Following PCR, samples were cleaned and normalized by using a Sequal Prep Normalization Plate (Life Technologies, Carlsbad, CA). Quality and quantity of the libraries were assessed using an Agilent DNA1000 chip and Qubit® dsDNA HS Assay Kit, respectively, and were standardized to 2 nM prior to pooling and sequencing. Sequencing was done with an Illumina MiSeq system (Illumina, San Diego, CA) by using Miseq reagent kit v. 3 (Illumina) to generate 2 × 300 bp paired end reads. Images were analyzed using the standard Illumina Pipeline, version 1.8.2.

### Sequence database processing and analyses

Illumina datasets were de-multiplexed by using MiSeq Reporter v2.2.31. (Illumina) with a Q20 minimum value as a quality filter, and then reads trimmed of forward and reverse primers by using cutadapt (Martin, 2011). For bacterial and archaeal libraries, paired sequences were merged into single reads by using FLASH (Magoč and Salzberg, 2011), and then length-filtered. For fungal libraries, the forward and reverse reads (300 bp each) were not large enough to establish physical overlap, so each of these libraries was processed individually. The Fungal ITS extractor was applied to fungal libraries to isolate ITS sequences by removing adjoining regions encoding ribosomal RNA (Nilsson et al., 2010). The QIIME (Quantitative Insights into Microbial Ecology) package (v. 1.8.0) was then utilized for generation of operational taxonomic units (OTU) by *de novo* clustering at 97% similarity. For bacterial and archaeal libraries, taxonomy was assigned by BLAST against GreenGenes (v. 2013_08) and the libraries were screened for chimeric reads by using Chimeraslayer against GreenGenes (v. 2013_08). For fungal libraries, the UNITE database (alpha version 2012_11) was used for taxonomic assignment of OTU by BLAST, and for chimera screening. In the fungal libraries, the quality and depth of coverage of forward reads was substantially greater than that of the reverse reads, thus libraries from the forward reads (i.e., ITS1) were used for further analysis of fungal communities.

The QIIME package (v. 1.8.0) was used for OTU-based analyses including rarefaction and computation of diversity metrics (excluding singletons). For comparisons between samples, libraries were rarefied to a common minimum number of 5000 sequences for each microbial group. Prism 6 (Graphpad, La Jolla, CA) was used to display the composition of each library by using average relative abundance for taxa believed to be best able to convey biologically relevant information. For example, proteobacterial classes were plotted individually rather than collectively at the phylum level because the classes have differing characteristics and showed varying abundance across the soils. For *Fungi*, genus-level taxa often dominated in soils, and thus these taxa were plotted. For congruence and uniformity, the taxa averaged in bar charts were used in the remainder of the analyses discussed below. Thus, community information from the replicate DNA extracts from single pooled soil samples was combined prior to statistical analyses. Primer-E v. 6 (PRIMER-E Ltd, Lutton, UK) was used for cluster analysis, non-metric multidimensional scaling (NMDS) ordination, similarity percentage (SIMPER) and RELATE, bioenvironmental step (BEST) analyses. For use in Primer-E executed routines, biotic data (taxa abundance) was square root-transformed, and then similarity matrices created based on Bray Curtis indices. For environmental data (edaphic characteristics), Euclidian distance-based similarity matrices were created with normalized data. The NMDS analysis (25 restarts, minimum Kruskal stress = 0.01) generated 2-D and 3-D plots, and the former were used for data presentation in the present report. Cluster analyses were used to generate similarity contours overlaid on NMDS plots. The SIMPER routine identifies the percent contribution of taxa to the average Bray-Curtis dissimilarity between samples (soils in the present study). The RELATE technique is a non-parametric form of Mantel test that generates a test statistic (Spearman Rho) gauging congruence between matrices of either biotic or abiotic data. For RELATE, 1000 random permutations were run to test two null hypotheses: (1) edaphic properties were not related to similarities in microbial community, and (2) there were no similarities between microbiome components among the soils (e.g., soils with relatively similar bacterial communities did not have similar archaeal or fungal communities). The BEST analysis, a multivariate non-parametric method, identified subsets of edaphic variables that yielded rank order similarities (Euclidean distance) between soils that best matched the rank order Bray-Curtis similarities derived from the microbial community composition (Clarke et al., 2008). Prior to use in BEST analysis, soil factors were normalized by subtracting the mean for a measurement, followed by division with the standard deviation for that measurement. Taxa abundance and was assessed for significant correlations with edaphic properties and false discovery rate (fdr) with the R programming environment (www.R-project.org).

### Sequence accession numbers

The data reported in this paper have been deposited in the NCBI Sequence Read Archive (http://www.ncbi.nlm.nih.gov/sra) under accession numbers: SRX1034830-SRX1034906.

## Results

### Characteristics of microbiome libraries

A total of 439,549,406 bacterial 16S rRNA gene sequences were obtained from all 27 amplicon libraries, generating 29,889 OTUs (Table S1). A total of 423,276 ITS1 sequences generated 1948 fungal OTUs (Table S2). Additionally, 1,735,424 archaeal sequences (268 OTUs) were retrieved from bacterial 16S rRNA gene sequences (Table S3). The archaeal- selective primers gave a total of 92,406,888 reads of which 36,800,815 were archaeal 16S rRNA gene sequences, and generated 2168 archaeal OTUs (Table S3). Thus, the depth of archaeal community interrogation was increased more than 20-fold over that obtained with the universal prokaryotic primers, yielding an eight-fold increase in archaeal OTU discovery. All rarefaction plots were rarefied to a common sampling depth of 5000 sequences (Figures S1–S3).

### Microbiome diversity and relation to soil characteristics

**Figure 1:**
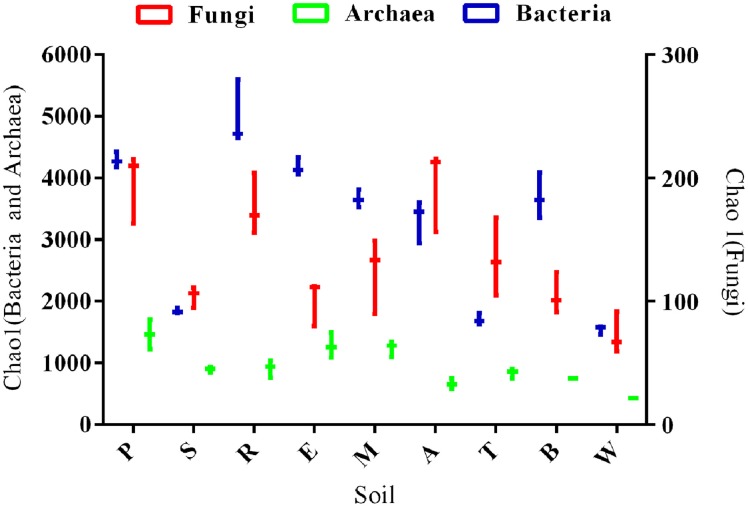
**Box and whisker plot of bacterial, fungal and archaeal species richness estimated by Chao1 metric**. All samples were rarified to a common read level. Letters indicate soil names, and are abbreviated as: A, Arena; B, Brasso; E, Ecclesville; M, Maracas; P, Piarco; R, River Estate; S, St. Augustine; T, Talparo and W, Princes Town.

In all soils, diversity (species richness) of soil microbiome components decreased in the order: *Bacteria* > *Archaea* > *Fungi*. Bacterial diversity was 2–7 times more than that of the *Archaea*, and 12–50 times greater than that of *Fungi* (Figure 1). Diversity of all microbiome components was highest in silt loam soils and lowest in the clays (Figure 1; Figures S1–S3), and showed significant negative correlations to: clay content (*Bacteria*: *p* = 0.0001, *r* = −0.4621; *Fungi*: *p* = 0.0259, *r* = −0.1902 and *Archaea*: *p* = 0.0206, *r* = −0.2401), Mg (*Bacteria*: *p* < 0.0001, *r* = −0.619; *Fungi*: *p* = 0.003, *r* = −0.307; *Archaea*: *p* = 0.033, *r* = −0.208) and Ca (*Bacteria*: *p* < 0.0001, *r* = −0.594; *Fungi*: *p* = 0.011, *r* = −0.239; *Archaea*: *p* = 0.018, *r* = −0.249). Bacterial and archaeal diversity also had significant negative correlations to pH (*p* = 0.0002, *r* = −0.455; *p* = 0.001, *r* = −0.410) whereas fungal diversity was not significantly correlated to pH (*p* = 0.094, *r* = 0.112).

### Composition of soil microbiomes

**Figure 2:**
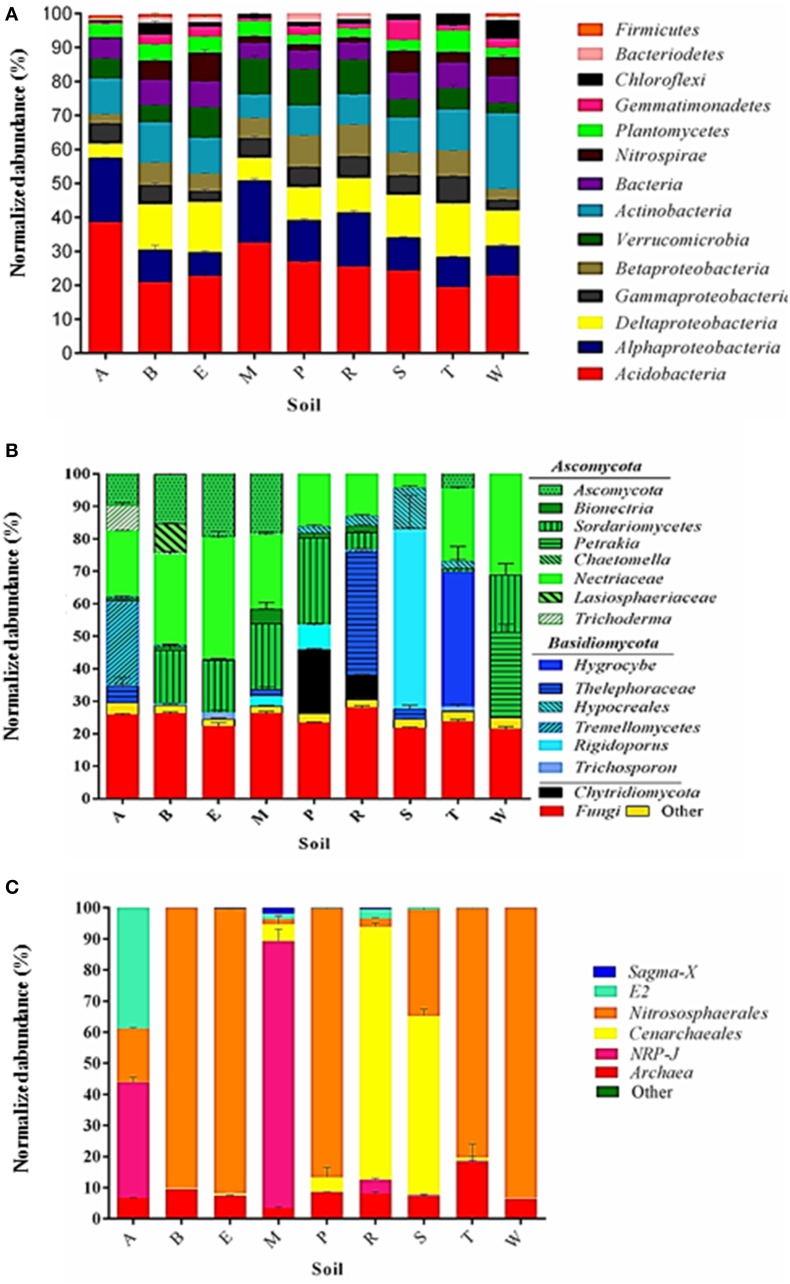
**Comprehensive view of the sequence content of soil libraries for bacteria (A), fungi (B), and archaea (C)**. Segments composing each bar are mean number of sequences in the indicated taxa normalized to the total number of sequences in each library. Standard error of each mean is indicated by lines within each segment. Letters indicate soil names and are abbreviated as: A, Arena; B, Brasso; E, Ecclesville; M, Maracas; P, Piarco; R, River Estate; S, St. Augustine; T, Talparo and W, Princes Town.

In the bacterial community, 13 phyla accounted for >96% of the sequence reads across all soils (Figure 2A; Table S1) with the majority being *Proteobacteria* (38%) and *Acidobacteria* (26%). Other phyla that comprised ≥3% of the bacterial communities were (Figure 2A): *Verrucomicrobia* (8%), *Actinobacteria* (6%), *Nitrospirae* (5%), *Planctomycetes* (5%), *Chloroflexi* (4%), and *Gemmantomindetes* (3%). A total of 2646 OTUs comprised the top quartile of the bacterial sequences, with the most prevalent OTUs across all soils identified as (fraction of reads composing top quartile) *Koribacteraceae* (25%) or *Nitrospirales* (11%). Fungal communities in six of nine soils were composed primarily of *Ascomycota* (Figure 2B), with the remainder of soils dominated by either *Basidiomycota* (River Estate and St. Augustine) or by unclassified fungi (Maracas).

A large group of sequences was assignable only to the domain level as *Fungi* (39%, 671 OTUs, Table S3). Manual BLAST-N against Genbank of representative sequences of OTU identified by UNITE as *Fungi* returned hits to a variety of genera, which were most often in the group referred to as “little brown mushrooms” (LBM). The LBM are so named because of difficulty in their identification, which is reflected in a high level of inconsistency in the genera returned as matching any given ITS sequence. The top quartile of all fungal sequences was composed of just seven OTUs. Of the seven OTUs, three were identified only as *Fungi* (i.e., LBM), the rest were species of *Ascomycota* (*Chaetomella* sp.) or *Basidiomycota* (*Trichosporon coprophilum, Rigidoporus microporus*, and *Thelephoraceae* sp.). Unlike bacteria, none of the major fungal OTUs were predominant across all the libraries.

Archaeal communities in eight of the nine soils were dominated by a single order either *Nitrososphaerales* or *Cenarchaeales* (Figure 2C). Notably, while reads assigned to the Cenarcheaotal order NRP comprised a relatively small fraction of the total library (10%) they accounted for 85% of the sequence reads in the Maracas Loam, and 30% in the Arena Sand (Figure 2C). The Arena sand was also distinctive as it was the only soil in which a single archaeal order was not dominant, and in which the *Euryarchaeota* were a major component, specifically the *Thermoplasmata* (Figure 2C). The only other soils in which *Euryarchaeota* were >1% were the Maracas and River Estate soils.

### Similarity of microbiome components among soils

**Figure 3:**
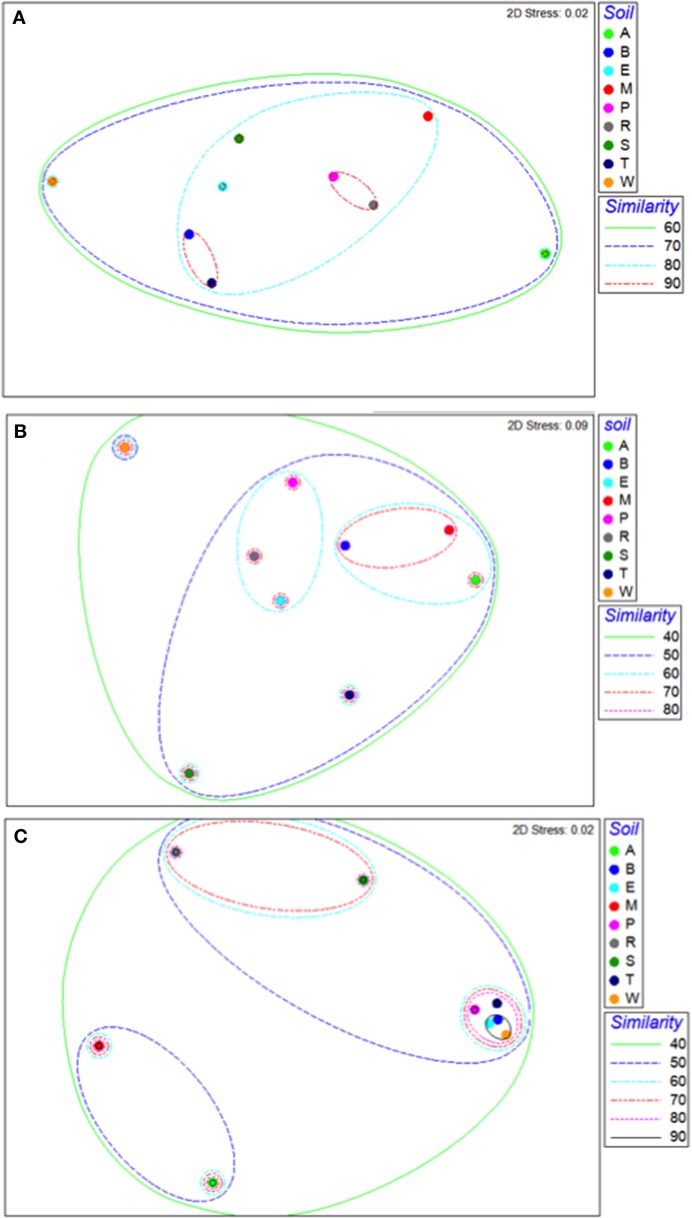
**Non-metric multidimensional scaling ordinations for bacterial (A), fungal (B), and archaeal (C) communities**. Letters indicate soil names, and are abbreviated as: A, Arena; B, Brasso; E, Ecclesville; M, Maracas; P, Piarco; R, River Estate; S, St. Augustine; T, Talparo and W, Princes Town. Numbers following letters indicate replicate number. Dotted lines are similarity contours derived from cluster analysis.

Bacterial communities in all soils were 70% similar, and most of these communities, except those of the Arena Sand and Princes Town clay, were 80% similar (Figure 3A). Within individual soils, bacterial communities were 90% similar (Figure 3A). The bacterial communities in the Piarco silty loam and River Estate loam were most alike, and clustered as closely as replicates of other soils, while those in the Arena sand and Princes Town clay were most dissimilar, and segregated to opposite ends of the NMDS ordination (Figure 3A). For fungal communities, the similarity across all soils was 40%, and the maximum similarity in fungal communities between soils was 60% (Figure 3B). Fungal communities of one soil segregated from the rest; that was Princes Town clay as was the case with *Bacteria* also (Figure 3B). The similarity of fungal communities within individual soils ranged from 50 (Princes Town clay, Figure 3B) to 90% (Piarco Silt loam, Figure 3B). Archaeal communities were 40% similar overall, and had a maximum degree of similarity of 80–90% between soils as well as within soils (Figure 3C). Mantel tests (RELATE) comparing microbiome components between soils yielded significant Rho-values for bacterial vs. archaeal communities (Rho = 0.58, *p* = 0.002), but not bacterial vs. fungal communities (Rho = 0.27, *p* = 0.128) or bacterial vs. fungal communities (Rho = 0.147, *p* = 0.25).

**Figure 4:**
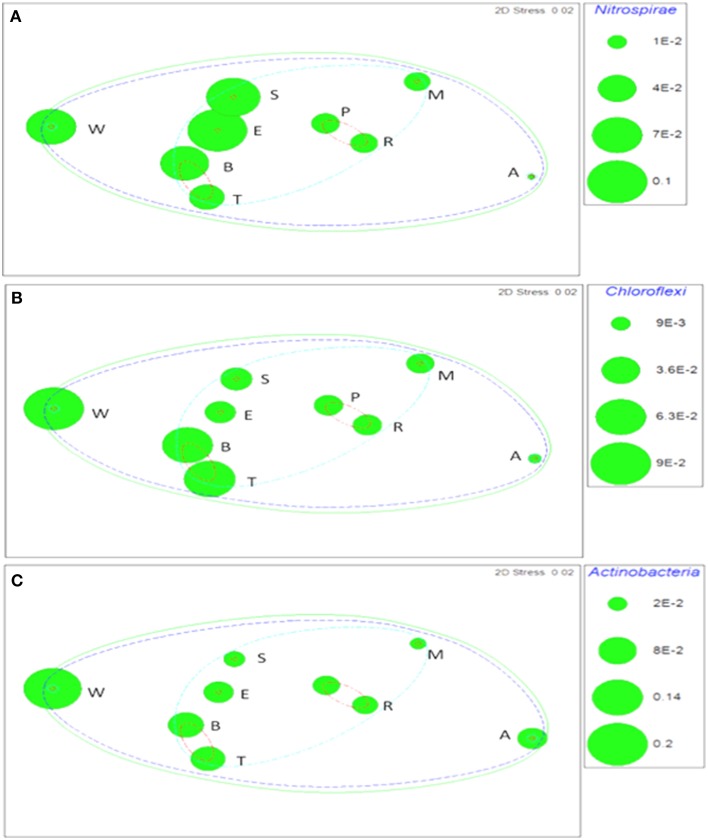
**Bubble plots of taxa discriminating soil bacterial communities: *Nitrospirae* (A) *Chloroflexi* (B), and *Actinobacteria* (C)**. Bubble areas are proportional to relative abundance (square root-transformed) of the indicated taxon. Dotted lines are similarity contours derived from cluster analysis and are color-coded as described for Figure 3. Letters are soil names abbreviated as: A, Arena; B, Brasso; E, Ecclesville; M, Maracas; P, Piarco; R, River Estate; S, St. Augustine; T, Talparo and W, Princes Town. Numbers following letters indicate replicate number.

**Figure 5:**
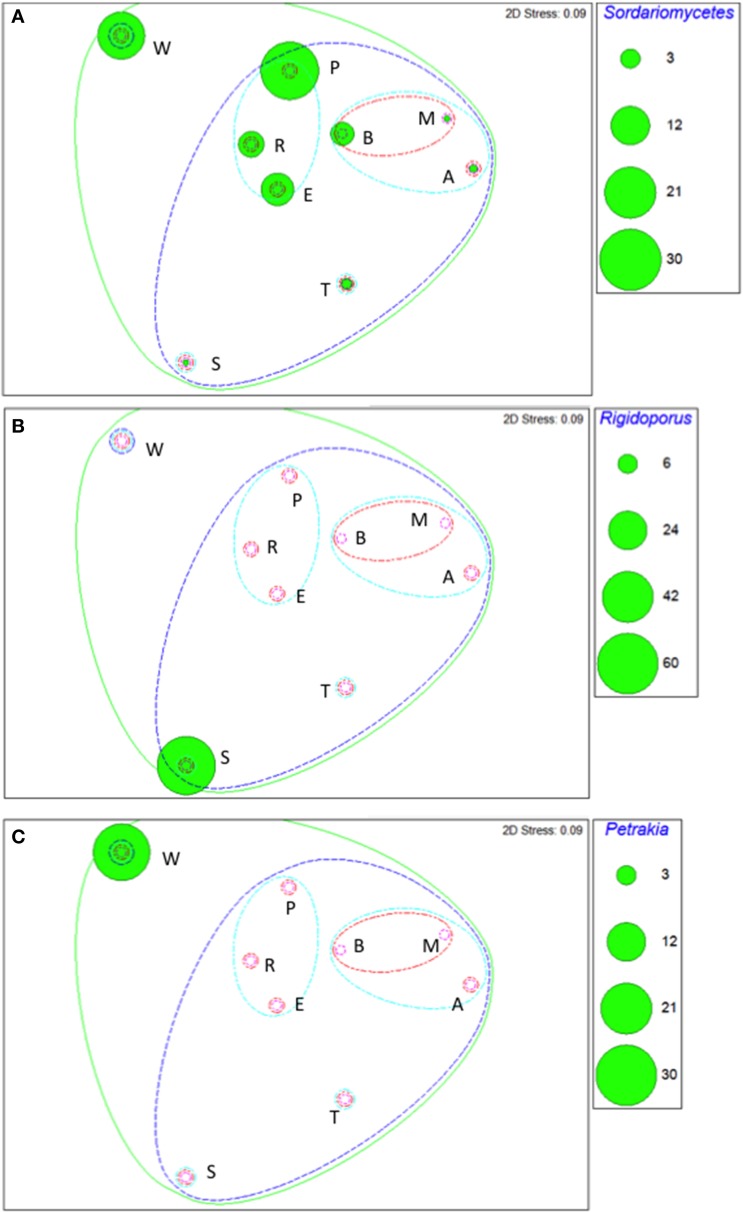
**Bubble plots of taxa discriminating soil fungal communities: *Sodariomycetes* (A), *Rigdoporous* (B), and *Petrakia* (C)**. Bubble areas are proportional to relative abundance (square root-transformed) of the relevant taxon. Dotted lines are similarity contours derived from cluster analysis and are color-coded as described for Figure 3. Letters indicate soil names and are abbreviated as: A, Arena; B, Brasso; E, Ecclesville; M, Maracas; P, Piarco; R, River Estate; S, St. Augustine; T, Talparo and W, Princes Town. Numbers following letters indicate replicate number.

**Figure 6:**
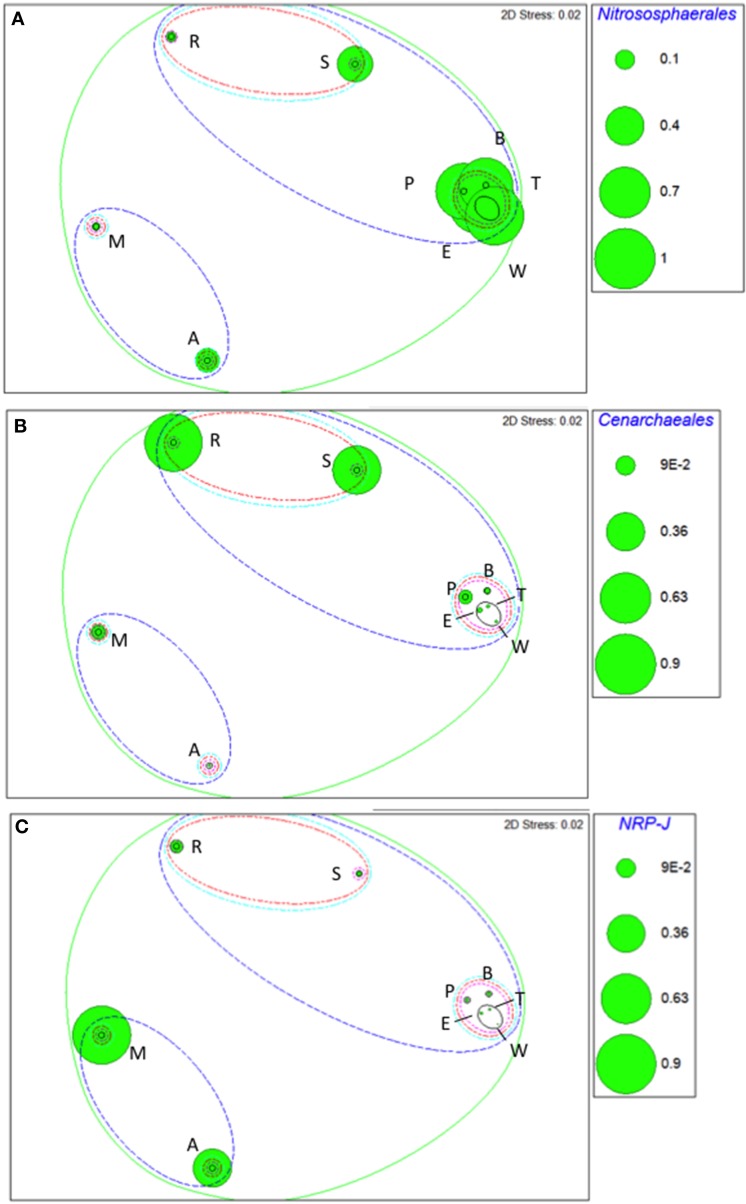
**Bubble plots of taxa discriminating soil archaeal communities: *Nitrososphaerales* (A), *Cenarchaeales* (B), and *NRP-J* (C)**. Bubble areas are proportional to relative abundance (square root-transformed) of the relevant taxon. Dotted lines are similarity contours derived from cluster analysis and are color-coded as described for Figure 3. Letters indicate soil names and are abbreviated as: A, Arena; B, Brasso; E, Ecclesville; M, Maracas; P, Piarco; R, River Estate; S, St. Augustine; T, Talparo and W, Princes Town. Numbers following letters indicate replicate number.

Taxa that defined differences between soils were identified by SIMPER, and displayed as bubble plots superimposed on NMDS ordinations (Figures 4–**6**). In bacterial communities, *Chloroflexi* and *Nitrospirae* were significant in distinguishing the two most dissimilar soils, Princes Town clay and Arena sand (Figures 4A,B). For other soils, variation in *Actinobacteria* abundance was a distinguishing feature (Figure 4C). For *Fungi*, the two most dissimilar soils, the Princes Town clay and the St. Augustine loam, were distinguished by *Petrakia* and *Rigidoporus*, respectively (Figures 5A,B). For *Archaea, Nitrososphaerales* was a constituent of all archaeal communities, but its abundance was a defining factor for the soils that clustered at 80–90% similarity in archaeal communities (Figure 6A). A key group for the Maracas and Arena soils was NRP-J, while *Cenarchales* was distinguishing element for the River Estate and St. Augustine soils (Figures 6A–C).

### Edaphic characteristics and microbiome components

Bacterial community composition had a relatively strong relationship to edaphic factors (Rho = 0.725, *p* < 0.001), while that of *Archaea* (Rho = 0.345, *p* < 0.002) and *Fungi* (Rho = 0.267, *p* = 0.001) were comparatively weak, but still significant. Rho-values that approached 1 indicated increasing strength of relation between edaphic factor(s) and variation in microbial community structure. For *Bacteria*, the edaphic characteristics that best explained the variation between soils in community structure were the combination of total carbon, sodium, magnesium and zinc (Rho = 0.834, *p* < 0.001). Soil factors best explaining fungal community variation among the soils were sodium, magnesium, phosphorus, boron and cabon to nitrogen ratio (Rho = 0.632, *p* < 0.001). For *Archaea*, BEST analysis yielded two sets of edaphic factors of equal strength (correlation 0.632, *p* < 0.001) that correlated with community variation, one with three variables (sulfur, sodium, and ammonium-N) and the other with four variables (clay, potassium, ammonium-N, and nitrate-N). Sodium level was the only edaphic factor that occurred in correlations with all three communities.

### Taxa abundance as a function of soil properties

Abundance of six bacterial taxa at either the class or phyla level (Table S4) was most extensively correlated with edaphic properties (number of correlations at *p* < 0.0001): *Acidobacteria* (15 correlations), *Deltaproteobacteria* (13 correlations) and *Nitrospiraea* (12 correlations), *Verrucomicrobia* (11correlations), *Chloroflexi* (11correlations), and *Alphaproteobacteria* (10 correlations). For *Acidobacteria*, the most significant positive correlations were to sand content (*p* < 0.0001, *r* = 0.759) and bulk density (*p* < 0.0001, *r* = 0.648), and significant negative correlations were to sodium (*p* < 0.0001, *r* = 0.690); clay (*p* < 0.0001, *r* = 0.551); potassium (*p* = 0.0168, *r* = −0.214), zinc (*p* = 0.0168, *r* = −0.590) and pH (*p* = 0.0168 *r* = −0.208). Correlations of *Alphaproteobacteria* abundance to edaphic properties mirrored the *Acidobacteria*, and both groups were significantly more abundant in the Arena sand than in all other soils. *Deltaproteobacteria* abundance was correlated to the same set of edaphic characteristics as the *Alphaproteobacteria*, except these correlations were all negative, and directly opposite to the pattern of *Alphaproteobacteria*. *Nitrospiraea* had significant positive correlations (*p* < 0.0001) with all soil nitrogen properties except ammonium. Notably, pH was significant for just 5 of 13 phyla, and was positively correlated with abundance of *Chloroflexi* (*p* < 0.0001, *r* = 0.549), *Actinobacteria* (*p* = 0.0005, *r* = 0.391), *Verrucomicrobia* (*p* < 0.0001, *r* = 0.452), and *Firmicutes* (*p* = 0.023, *r* = 0.189). The *Acidobacteria* were negatively correlated with pH (*p* = 0.0168, *r* = −0.208).

For fungi (Table S5) certain genera were important eg. *Trichoderma* abundance had the largest number of significant correlations to edaphic characteristics (11), and correlated positively to bulk density (*p* = 0.0047, *r* = 0.278) and sand content (*p* = 0.001, *r* = 0.351) and negatively to clay content (*p* = 0.0427, *r* = −0.154), total organic carbon (*p* = 0.0147, *r* = −0.215), total nitrogen (*p* = 0.028, *r* = −0.177), silt (*p* = 0.026, *r* = −0.182) and to all the other elements examined except Mg (Table S5). None of the fungal taxa showed significant correlations in their abundance to pH.

Among archaeal taxa (Table S6), meaningful differences were at the order *Euryarcheota* (E2), had the greatest number of significant correlations to soil characteristics (eight) followed by NRP-J (seven). Group E2 had significant positive correlations to bulk density (*p* = 0.0046, *r*^2^ = 0.403), sand (*p* = 0.0002, *r* = 0.5948) and significant negative correlations to total organic carbon (*p* = 0.0109, *r* = −0.341), total nitrogen (*p* = 0.0196, *r* = −0.296), silt (*p* = 0.0032, *r* = −0.088), sodium (*p* = 0.0037, *r* = −0.418), zinc (*p* = 0.031, *r* = −0.259) and sulfur (*p* = 0.0477, *r* = −0.223). The *Nitrososphaerales* had significant positive correlations to phosphorus (*p* = 0.0011, *r*^2^ = 0.454), organic carbon (*p* = 0.0494, *r* = 0.154), total nitrogen (*p* = 0.026, *r* = 0.181), and total Kjeldahl nitrogen (*p* = 0.0007, *r* = 0.15) and a significant negative correlation to pH (*p* = 0.0227, *r* = −0.1909). The *Cenarchaeales* were the only archaeal taxon that showed no significant correlations to soil properties.

## Discussion

Bacteria were consistently the most diverse component of the soil microbiomes, and species richness in these tropical soils was within the range of that determined for soils from other biomes (Roesch et al., 2007; Nacke et al., 2011; Li et al., 2014; Tripathi et al., 2014). Soil fungal diversity estimated from other NGS analyses of ITS1 ranges from 20 to 60 OTUs (Shi et al., 2014) to >2000 OTU (Buée et al., 2009). Thus, fungal species richness in the tropical soils examined here, was an intermediate level, and similar to that reported for Mediterranean soils (Orgiazzi et al., 2013). In the present study, archaeal diversity was comparatively high, which contrasts to prior NGS studies reporting relatively low diversity of soil archaeal communities. A global survey of soils revealed just two archaeal OTUs (Bates et al., 2011), archaeal communities in soil of Antarctic dry valleys were composed of 10 OTUs (Richter et al., 2014) and those in Malaysian forest and non-forest soils contained 30–60 OTUs (Tripathi et al., 2013).

Microbiome components contrasted in species turnover patterns, with the composition of bacterial communities being more similar between soils than were those of archaeal or fungal communities. For *Bacteria*, changes in abundance of taxa distinguishing soils were matters of degree and not presence vs. absence, which was often the case for *Fungi* and *Archaea*. For example, while *Chloroflexi* and *Nitrospirae* were present in all soils, these taxa distinguished the Arena and Princes Town soils from the others because of relatively higher abundance. In contrast, with *Fungi, Petrakia* was highly abundant in the Princes Town clay, but absent from other soils. Similarly, archaeal communities were distinguished by NRPJ, E2 and *Cenarchaea*, which were dominant or major components in certain soils, but minor or absent in others. Although species turnover patterns of archaea differed from those of bacteria, the community structures of these two groups were correlated across soils. In contrast, fungal community structure varied independently of the other two microbiome components and was not correlated to that of either bacteria or archaea. Thus, soils having relatively similar bacterial communities could be predicted to house similar archaeal communities, they could not be predicted to have similar fungal communities.

For all three microbial groups, variation in community structure was significantly correlated with edaphic factors, but the linkage was stronger with *Bacteria* than with *Fungi* or with *Archaea*. For the latter two groups, a weaker connection suggests that variables other than soil characteristics were important in determining community structure. Prior investigators have also observed soil characteristics to correlate weakly with fungal community structure, and in some of cases, it was more strongly correlated to plant type or diversity (Zinger et al., 2011; Orgiazzi et al., 2013). The relatively weak correlation of edaphic properties with archaeal community structure was somewhat surprising, as their prokaryotic lifestyle, like that of the *Bacteria*, might be presumed to establish a strong linkage with soil physicochemical properties, which would be reflected in soil characteristics being the predominant factor affecting community structure. However, the present data suggest that unknown factor(s) were as strong, or stronger, than edaphic characteristics in shaping archaeal communities.

While the overall strength to which edaphic characteristics were correlated with community structure varied with microbiome components, all microbial groups nonetheless had significant correlations with soil properties. But, the hypothesis that pH would be the predominant edaphic variable affecting the soil microbiome was not supported. In contrast, mineral characteristics related to texture and elemental composition predominated as factors affecting diversity, taxa abundance and microbiome structure. Textural characteristics are often correlated with microbial community characteristics, and have been interpreted to reflect effects translated through a variety of physicochemical pathways such as nutrient status, aeration or moisture holding capacity. Relationships between soil microbiomes and elemental levels have not been widely explored, but in a global survey of soil fungi, calcium was one of the strongest predictors of fungal species richness, and the effect theorized to reflect impacts of calcium on metabolic processes or soil organic matter decomposition (Tedersoo et al., 2014). In the present study, sodium level was a factor affecting all microbiome components, but mechanisms by which it might have directly affected microbes were not readily apparent, as concentrations were within a range sufficient to provide the small amounts needed in microbial metabolism, but below levels that might impart deleterious effects *via* solute stress. Soil sodium levels were probably more important to the vegetation, as that element has a key role in chlorophyll synthesis and carbon fixation by C4 grasses, which were the dominant species in all locations except the Arena forest soil (Subbarao et al., 2003). Consequently, it's possible that the sodium effect on microbial communities was partly, if not primarily, indirect and transmitted *via* effects on plant growth.

Relatively little is known about tropical soil microbiomes, and the present study provided insights into unique characteristics of that biosphere. For example, a notable aspect of the bacterial community was a prevalence of OTUs affiliated with organisms predicted to exhibit anaerobic and/or (photo)lithoautotrophic growth such as aerobic phototrophic *Chloracidobacteria*, anaerobic phototrophic *Rhodoplanes* and the predominantly anaerobic sulfate-reducing *Syntrophobacterace* (Loy et al., 2004; Bryant et al., 2007; Lin et al., 2014). Also, while *Nitrospirae* (predominantly *Nitrospirales*) represented a large fraction of the bacterial sequences, no reads were assigned as *Nitrobacter*, which may indicate chemolithotrophic nitrite oxidation in these soils may be predominantly mediated by *Nitrospira*-like organisms rather than by alphaproteobacterial *Nitrobacter* types, such as been determined in other soils by probing with ^13^CO_2_ (Daebeler et al., 2014). The composition of fungal communities in these tropical soils varied substantially from that reported for typical abundances in a global survey of soils (Tedersoo et al., 2014) in terms of predominant phyla and orders. Fungi mediating carbon cycling in tropical forests are not well known, and the high abundance in the Arena forest soil of *Trichosporon* (notable for cellulose and hemicellulose degradation (König et al., 2013) may provide insight into this process. For *Archaea*, abundance of *Cenarchaeales* in some soils was novel, as these organisms are known mostly from marine systems. The Arena forest soil was notable for the high composition of *Euryarchaeota*, specifically *Thermoplasmata* order E2, which is not commonly a major component of soil archaeal communities. While the E2 group was originally associated with high temperature environments, it has since been identified in non-extreme habitats (Jones et al., 1998; Saiz-Jimenez and Laiz, 2000), but metabolic characteristics of these organisms remain unknown.

## Conclusions

Understanding the microbial biodiversity that exists in soils, and how it varies as a function of soil characteristics, is key to elucidating associations between soil ecosystem function and the microbiome that sustains it. Much attention has centered on *Bacteria*, but results of the present study underscore the need for focused exploration of *Fungi* and *Archaea*. While each of the three component communities differed in species turnover patterns, soils having relatively similar bacterial communities also housed similar archaeal communities. In contrast, the composition of fungal communities had no correlation to bacterial or archaeal communities. Although interactions between communities undoubtedly occur, the picture emerging from the present study is one in which evolution of fungal and prokaryotic components of the microbiome may occur along separate paths. Finally, in future soil microbiome studies, elemental speciation analyses could yield potential mechanistic interpretations of correlations between soil elemental composition and microbial community structure. While there is a great focus on pH, this study showed that factors other than pH were more important than or as important as pH.

### Conflict of interest statement

The authors declare that the research was conducted in the absence of any commercial or financial relationships that could be construed as a potential conflict of interest.

## Supplementary material

Figure S1 **Rarefaction curves for bacterial amplicon libraries**. A, Arena; B, Brasso; E, Ecclesville; M, Maracas; P, Piarco; R, River Estate; S, St. Augustine; T, Talparo and W, Princes Town.

Figure S2 **Rarefaction curve for fungal amplicon libraries**. A, Arena; B, Brasso; E, Ecclesville, M, Maracas; P, Piarco; R, River Estate; S, St. Augustine; T, Talparo and W, Princes Town.

Figure S3 **Rarefaction curve for archaeal amplicon libraries**. A, Arena, B, Brasso, E, Ecclesville, P, Piarco, R, River Estate, S, St. Augustine, T, Talparo and W, Princes Town.

Table S1 **Bacterial library across soils**.

Table S2 **Fungal library across soils**.

Table S3 **Archaeal library across soils**.

Table S4 **Bacterial taxa abundance as a function of soil properties**.

Table S5 **Fungal taxa abundance as a function of soil properties**.

Table S6 **Archaeal taxa abundance as a function of soil properties**.
