# Supplementary figures and images for: Relations of microbiome characteristics to edaphic properties of tropical soils from Trinidad
Source: Front Microbiol. 2015 Sep 30;6:1045. doi: 10.3389/fmicb.2015.01045 (PMC4588118; doi:10.3389/fmicb.2015.01045)

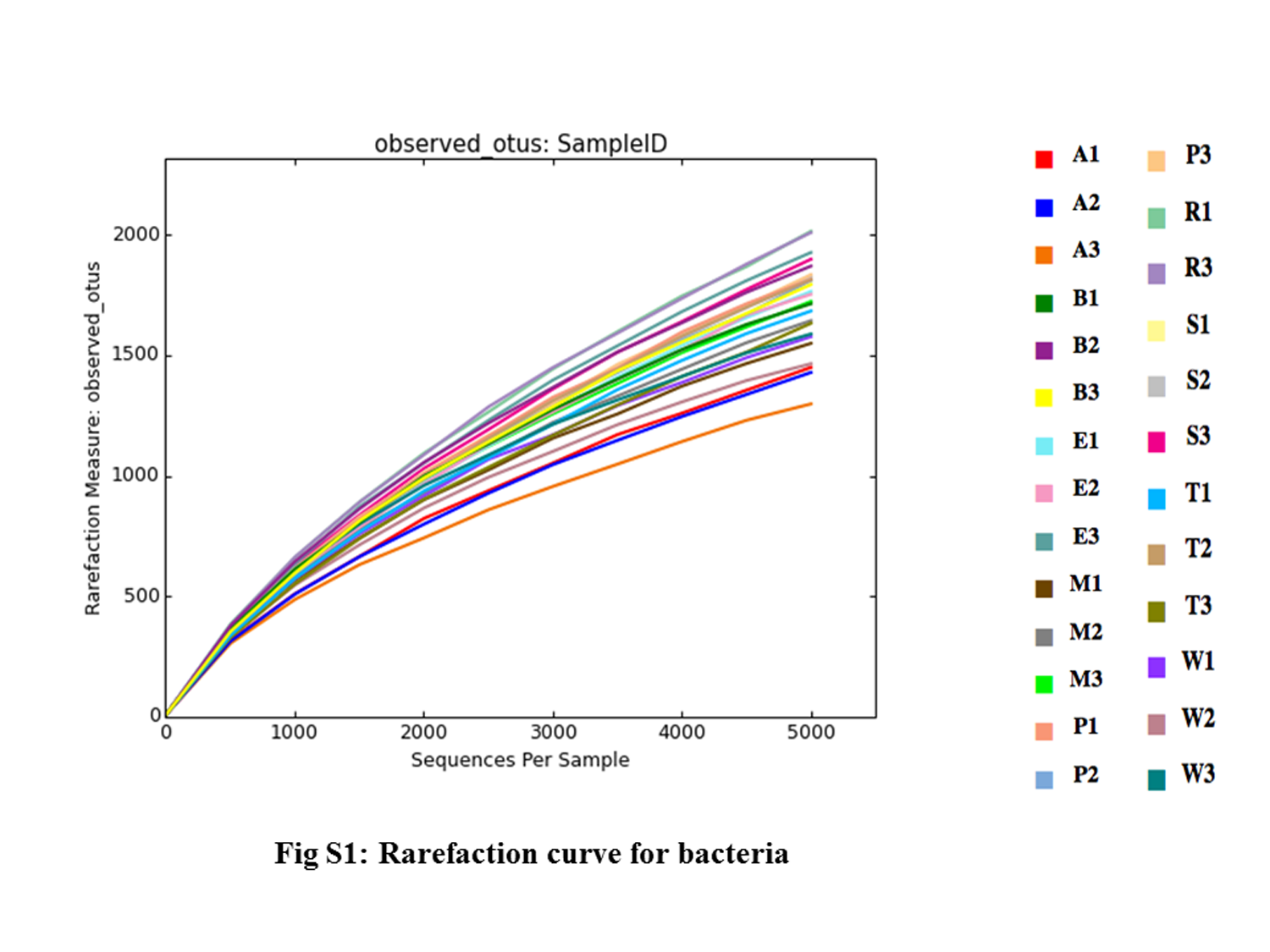

Supplement: Figure S1 — Rarefaction curves for bacterial amplicon libraries. A, Arena; B, Brasso; E, Ecclesville; M, Maracas; P, Piarco; R, River Estate; S, St. Augustine; T, Talparo and W, Princes Town. [file Image1.TIF]

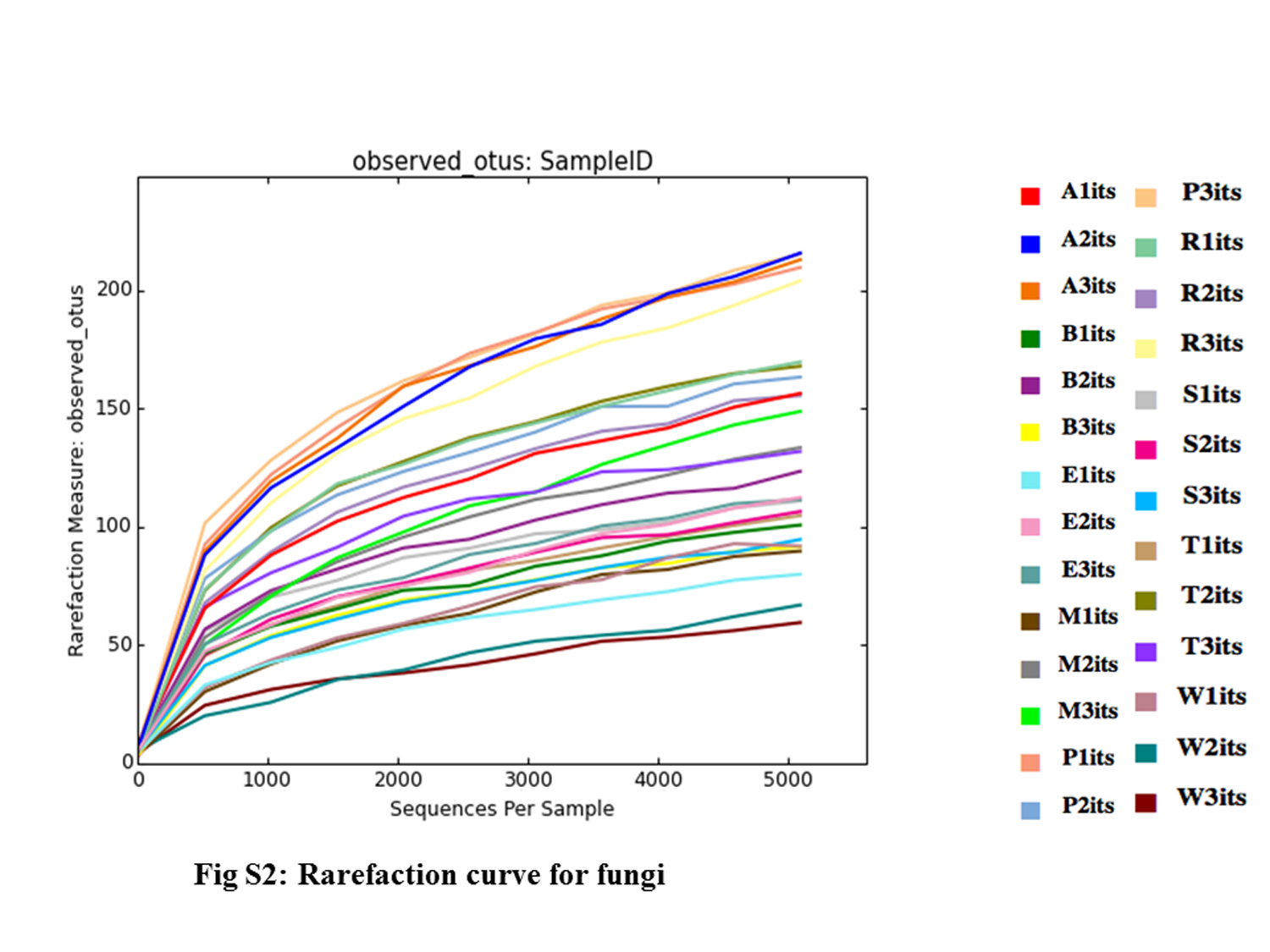

Supplement: Figure S2 — Rarefaction curve for fungal amplicon libraries. A, Arena; B, Brasso; E, Ecclesville, M, Maracas; P, Piarco; R, River Estate; S, St. Augustine; T, Talparo and W, Princes Town. [file Image2.TIF]

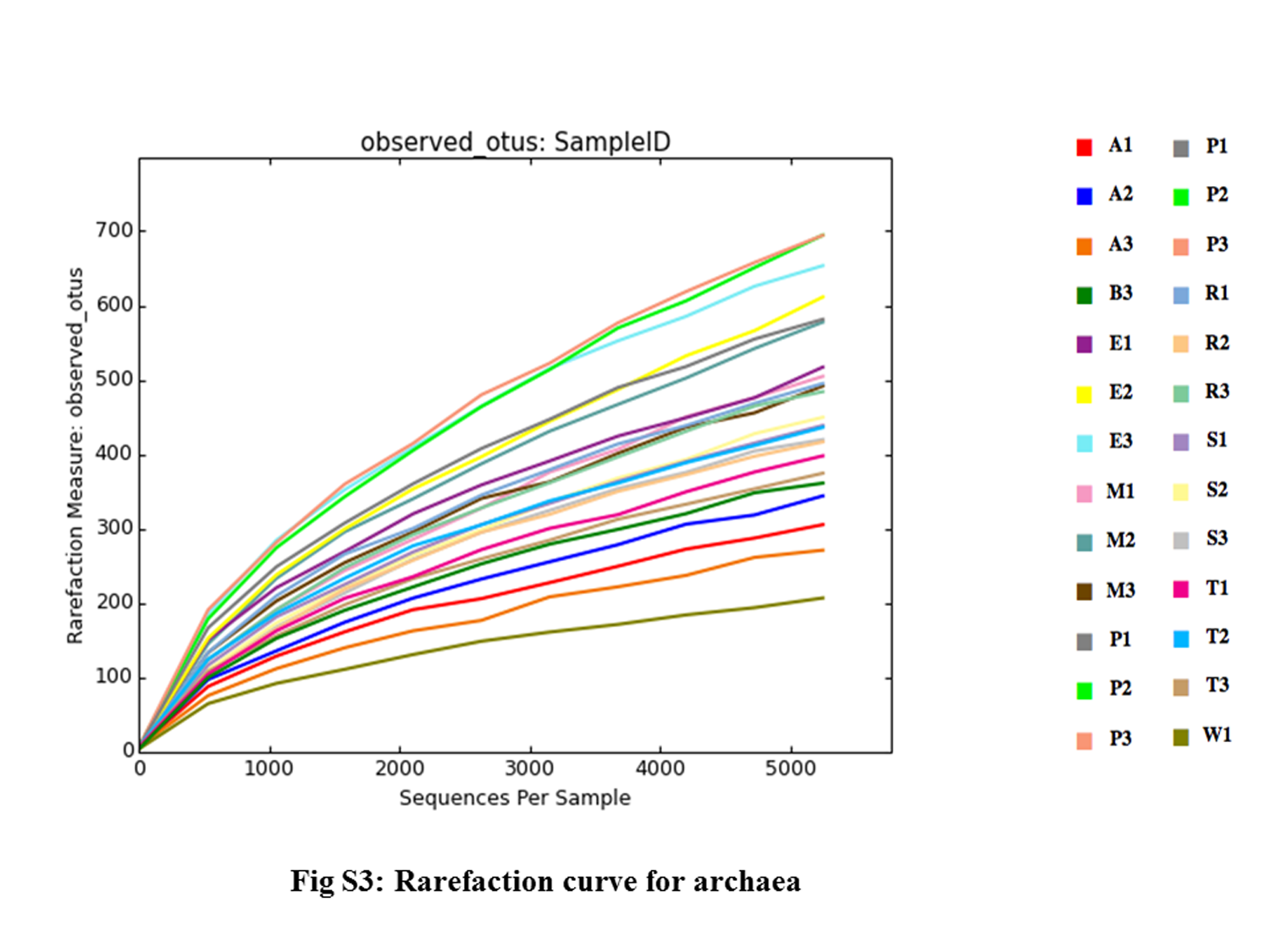

Supplement: Figure S3 — Rarefaction curve for archaeal amplicon libraries. A, Arena, B, Brasso, E, Ecclesville, P, Piarco, R, River Estate, S, St. Augustine, T, Talparo and W, Princes Town. [file Image3.TIF]
